# Detecting hand joint ankylosis and subluxation in radiographic images using deep learning: A step in the development of an automatic radiographic scoring system for joint destruction

**DOI:** 10.1371/journal.pone.0281088

**Published:** 2023-02-13

**Authors:** Keisuke Izumi, Kanata Suzuki, Masahiro Hashimoto, Toshio Endoh, Kentaro Doi, Yuki Iwai, Masahiro Jinzaki, Shigeru Ko, Tsutomu Takeuchi, Yuko Kaneko

**Affiliations:** 1 Department of Internal Medicine, Division of Rheumatology, Keio University School of Medicine, Tokyo, Japan; 2 Medical AI Center, Keio University School of Medicine, Tokyo, Japan; 3 Division of Rheumatology, National Hospital Organization Tokyo Medical Center, Tokyo, Japan; 4 Fujitsu Limited, Kanagawa, Japan; 5 Department of Radiology, Keio University School of Medicine, Tokyo, Japan; 6 Department of Systems Medicine, Keio University School of Medicine, Tokyo, Japan; Korea National University of Transportation, KOREA, REPUBLIC OF

## Abstract

We propose a wrist joint subluxation/ankylosis classification model for an automatic radiographic scoring system for X-ray images. In managing rheumatoid arthritis, the evaluation of joint destruction is important. The modified total Sharp score (mTSS), which is conventionally used to evaluate joint destruction of the hands and feet, should ideally be automated because the required time depends on the skill of the evaluator, and there is variability between evaluators. Since joint subluxation and ankylosis are given a large score in mTSS, we aimed to estimate subluxation and ankylosis using a deep neural network as a first step in developing an automatic radiographic scoring system for joint destruction. We randomly extracted 216 hand X-ray images from an electronic medical record system for the learning experiments. These images were acquired from patients who visited the rheumatology department of Keio University Hospital in 2015. Using our newly developed annotation tool, well-trained rheumatologists and radiologists labeled the mTSS to the wrist, metacarpal phalangeal joints, and proximal interphalangeal joints included in the images. We identified 21 X-ray images containing one or more subluxation joints and 42 X-ray images with ankylosis. To predict subluxation/ankylosis, we conducted five-fold cross-validation with deep neural network models: AlexNet, ResNet, DenseNet, and Vision Transformer. The best performance on wrist subluxation/ankylosis classification was as follows: accuracy, precision, recall, F1 value, and AUC were 0.97±0.01/0.89±0.04, 0.92±0.12/0.77±0.15, 0.77±0.16/0.71±0.13, 0.82±0.11/0.72±0.09, and 0.92±0.08/0.85±0.07, respectively. The classification model based on a deep neural network was trained with a relatively small dataset; however, it showed good accuracy. In conclusion, we provided data collection and model training schemes for mTSS prediction and showed an important contribution to building an automated scoring system.

## Introduction

Rheumatoid arthritis (RA) is an inflammatory disease of the joints, in which the joints are destroyed as the disease progresses. In the practice of RA, the evaluation of joint destruction is important. Among rheumatic diseases, RA is the most common, and joint destruction affects the daily activities and quality of life of patients. One of the unique tasks in diagnosing RA is estimating the van der Heijde-modified total Sharp score (mTSS [1]) based on X-ray images. Clinical studies widely use mTSS as a quantitative evaluation of joint destruction. However, mTSS has some challenges: the evaluation requires skill, time, and effort; in addition, the scores given by the evaluators are not consistent. Consequently, mTSS is not used in daily practice because it is cumbersome, and an automatic radiographic scoring system is required.

Image recognition technology using deep neural networks (DNNs) has exceeded human performance in various tasks, such as classification [2], object detection [3], and object segmentation [4]. DNN can automatically extract image features in its middle layers which enables it to handle high-dimensional images without complicated preprocessing. In the medical field, DNNs are beginning to be applied to the discrimination of diabetic retinopathy, skin cancer, gastrointestinal cancer, etc. [5–9], and are expected to be an alternative to diagnostic work.

In rheumatic diseases, DNNs are used in some studies. Burlina et al. predicted myositis from ultrasound images of muscles [10]. Lin et al. predicted methotrexate liver damage from electronic medical records [11]. Raddy et al. predicted readmission within 30 days of systemic lupus erythematosus discharge [12]. As in other medical fields, the use of data from various domains such as ultrasound images and medical records is increasing. This study applies DNNs to the rheumatic diagnosis of subluxation and ankylosis using X-ray images and verifies their effectiveness.

There is a study that predicts mTSS end-to-end by combining two DNNs that predict joint areas and their scores [13]. The study used a single X-ray image and a different procedure from the normal mTSS derivation. The results showed a low score. We aim to predict joint subluxation and ankylosis, which is part of the mTSS procedure, and achieve high accuracy within a limited scope as a first step in the development of an automatic radiographic scoring system for bone destruction. Subluxation/ankylosis prediction is important in improving the accuracy of mTSS because subluxation and ankylosis are given a large score in mTSS.

In this study, we trained DNNs end-to-end to predict wrist subluxation and ankylosis on X-ray images. We developed dedicated annotation tools for RA to efficiently collect the data needed to train the model. Our work is the first step in developing an automatic radiographic scoring system for bone destruction in X-ray images using deep learning. The proposed model contributes toward improving mTSS accuracy. Finally, we discuss the model behavior by visualizing the parts that contribute to the prediction result of the trained model.

## Method

### Patients and dataset

We randomly extracted 216 hand X-ray images acquired from patients who visited the rheumatology department of Keio University Hospital in 2015 from the electronic medical record system of the hospitas. One X-ray image included a pair of hands. Patients with RA who met the 1987 ACR classification criteria or the 2010 EULAR/ACR classification criteria were selected. This study protocol was approved by the Ethics Committee at Keio University School of Medicine (No. 20160316), and written informed consent was waived because of the retrospective study design.

We annotated subluxation/ankylosis in proximal interphalangeal (PIP), metacarpal phalangeal (MP), and wrist joints in the extracted X-ray images using our newly developed image labeling software (annotation tool) and based on the agreement between well-trained rheumatologist and radiologist. After annotation, 21 X-ray images containing one or more subluxation joints and 42 X-ray images with ankylosis were identified and used to train the DNNs.

### Annotation tool

**Fig 1:**
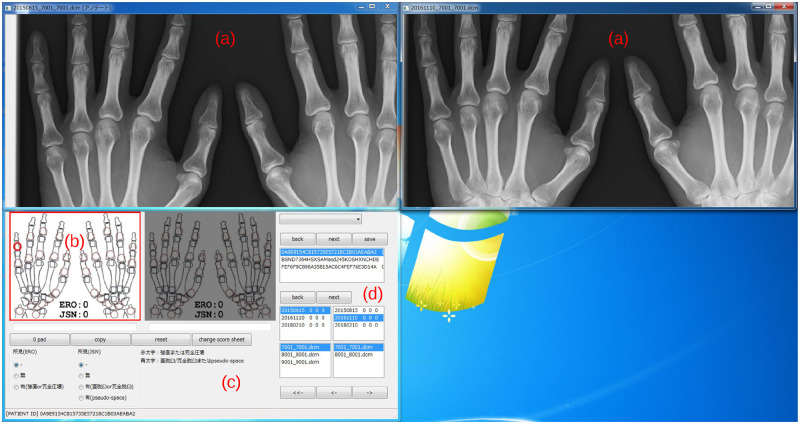
Developed annotation tool for automatic radiographic scoring system. Windows (a) are the image windows that show X-ray images to be annotated, and windows (b) to (d) are the annotation input windows. Since our tool works on the electronic medical record system, the annotators annotated the images during their spare time.

In this study, we developed a dedicated annotation tool for mTSS [14]. Fig 1 shows an overview of the annotation tool. Since mTSS needs to be scored by comparing the images at two different times, the tool always displays two images. A window is displayed for each image, and the display screen can be enlarged, reduced, moved, and reset (Fig 1a). X-ray images were saved in DICOM format and displayed in 2010 × 1670 pixels and 1024 gradations. The image resolution was designed in consultation with rheumatologists to ensure their sufficiency for scoring mTSS.

The annotator input scores are shown on a dedicated mTSS screen. Since the X-ray images and input screens are displayed in separate windows, the annotators can choose the convenient layout to work with. The input window has a simple hand diagram that shows the current input location (Fig 1b), a finding selection screen (Fig 1c), and an input image selection screen (Fig 1d). The scoring order of the tool was set according to the mTSS rules. The annotators used either the mouse or keyboard to annotate.

Annotating medical images is usually very laborious. Healthcare professionals are so busy that they cannot do a lot of annotations during off-hours. However, since the amount of training data affects the performance of DNNs, we developed a system to make annotation work efficient. Our tool was designed to operate on the electronic medical record system. The annotators were able to perform annotation in their spare time and we efficiently collected the data for DNN training. Although there are some DNN techniques for small datasets such as semi-supervised learning and unsupervised learning [15–17], annotation tools built into the work are effective in continuously improving model performance. We plan to release our annotation tool in the future.

### Model

We conducted learning experiments with DNN models for image classification: AlexNet [2], ResNet [18], DenseNet [19], and Vision Transformer (ViT [20]). We identified a suitable model for RA X-ray image classification by comparing multiple models with different network structures. We briefly describe the four models used in our experiments. AlexNet is a model composed of multiple convolutional layers and is a pioneering model that has improved the performance of image recognition tasks. ResNet has a shortcut connection to learn the residual function that references the input of layers, rather than learning only the optimal output of layers. This makes it possible to perform training with deeper layers than the normal DNNs. DenseNet uses “Dense blocks,” in which all sub-blocks are densely skip-connected, as main components. While keeping the basic idea of ResNet, it has developed into a large-scale multi-layer model by increasing the number of residual connections. ViT is a model that uses Transformer [21] attracting attention in the natural language processing field. ViT does not use convolutional layers and treats image patches like words, thereby achieving high performance at a low computational cost.

**Fig 2:**
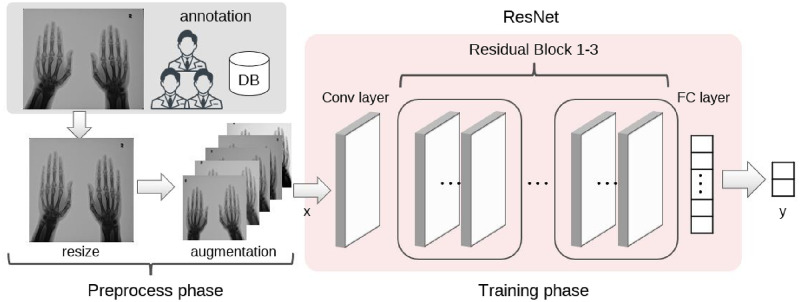
Overview of preprocessing and training phase of our method. Annotated images were resized and augmented before inputting to the DNN model. We trained two models to predict each label: subluxation and ankylosis. In the experiment, we also verified by replacing ResNet in the figure with several DNN models (AlexNet, DenseNet, ViT).

Fig 2 shows an overview of the entire training. In the experiment, the output number of the fully connected (FC) layer connected at the end of the model was changed to two to indicate the presence or absence of subluxation/ankylosis findings. In this study, the model was trained on the input image *x* for each binary classification of ankylosis and subluxation. For loss function *L*, we used Softmax cross-entropy, which is expressed by the following formula:

$$\begin{array}{c} L=-\sum_{t}\sum_{c}{\hat{y}}_{c}^{t}\text{log}y_{c}^{t}, \end{array}$$ (1)

where *t* is data number, *c* is the class number, *y* is the class probability vector by softmax function, and $\hat{y}$ is the teaching signal that is a one-hot vector. During model training, the parameters were optimized by minimizing *L*. The above loss function *L* is common to all models.

### Training setup

We performed five-fold cross-validation. The images were randomly divided into five datasets (Fold numbers 1 to 5). At this time, images with findings of subluxation/ankylosis were equally allocated to each dataset. Each fold dataset had approximately 172 training data samples and 43 test data samples. To train the DNN models, we used NVIDIA GeForce2070 as GPU, and each training took approximately 2–3 hours.

We used Adam [22] and AdamW [23] as the optimizers in the experiment and compared their performances. Adam is often used for training DNNs, and AdamW is an improved version of Adam. We used the parameters proposed in previous studies for each optimizer [22, 23]. Owing to the difference between natural and medical images, we did not use the weight parameters pre-trained with ImageNet [2]. The batch size was set to 64, and all models were trained for 500 epochs. Before inputting images to the model, the annotated image data were converted to grayscale bitmap format (244 × 244 pixels and 256 gradations). The values of all pixels of the image were normalized. The means for each channel were [0.485, 0.456, 0.406], and the standard deviations were [0.229, 0.224, 0.225]. To increase the robustness of the model, we performed some augmentations: brightness and saturation conversions, horizontal flipping, and random-position clipping. The final image input size was 224 × 224 pixels. During the evaluation phase, the images cropped from the center were used as the input.

### Evaluation and analysis

The trained models were evaluated in terms of accuracy, precision (synonymous with positive predicted value), recall (true positive rate, synonymous with sensitivity), specificity (true negative rate), and F value (harmonic average of precision and recall rate). We also calculated the area under the curve (AUC) of the receiver operating characteristic (ROC) curve. We conducted five training experiments with different seeds for each experimental setup: type of DNN model/dataset/optimizer. The above evaluation was performed for each trial.

In addition, we visualized the activated parts of the trained model using gradient-weighted class activation mapping (Grad-CAM [24]). Grad-CAM can calculate the contribution of the input image based on the gradient information of each DNN layer as follows.

$$\begin{array}{c} \alpha_{c}^{k}=\frac{1}{Z}\sum_{i}\sum_{j}\frac{\partial y_{c}}{\partial A_{i,j}^{k}}, \end{array}$$ (2)

$$\begin{array}{c} G_{c}=ReLU\left(\sum_{k}\alpha_{c}^{k}A^{k}\right), \end{array}$$ (3)

where *Z* is the number of pixels in the feature map, *k* represents the channel, and *i*, *j* represent the pixel position. The weight *α* of the feature map *A* is calculated from the gradient information obtained through backpropagation (Eq 2). The gradient, other than the output target class, is set to 0. By calculating the weighted sum of *α* and *A*, the contribution level *G*_*c*_ of the class *c* is calculated (Eq 3). Since it is difficult to evaluate the basis of judgment of the DNN model quantitatively, it is important to compare whether the model’s activation is consistent with the knowledge of the doctor. We discuss the trained subluxation/ankylosis classification model using these visualizations in the experimental section.

## Result and discussion

### Subluxation classification

Table 1 shows the classification results of wrist subluxation. As described in the previous section, we used four models, AlexNet, ResNet, DenseNet, and ViT, and two optimizers, Adam and AdamW. The 3rd–6th columns show the performances of the trained model, and the higher the number, the better the performance. Focusing on the average AUC, ResNet using Adam optimizer achieved the best performance. With the overall dataset, ResNet achieved the following averaged results: accuracy was 0.97±0.01, precision was 0.92±0.12, recall was 0.77±0.16, F1 Value was 0.82±0.11, AUC was 0.92±0.08. However, the performances of AlexNet and DenseNet are not bad, confirming that the convolutional layer-based model effectively recognizes rheumatism medical images. Also, there was no significant difference in the results between Adam and AdamW, and the optimizer difference did not seem to affect the model performance. ViT performed the worst among the four models. This is probably because the model was too large for the dataset and could not be generalized well.

**Table 1:** Results of subluxation classification.

Model	Optimizer	Dataset	Accuracy	Precision	Recall	F1 Value	AUC	Average AUC
AlexNet [2]	Adam [22]	1	0.93±0.03	0.50±0.50	0.35±0.37	0.40±0.43	0.74±0.26	0.80±0.17
		2	0.96±0.03	0.80±0.44	0.60±0.33	0.68±0.38	0.84±0.19	
		3	0.92±0.01	0.73±0.25	0.45±0.11	0.52±0.09	0.81±0.13	
		4	0.92±0.05	0.55±0.40	0.55±0.32	0.54±0.35	0.74±0.18	
		5	0.96±0.01	0.92±0.10	0.80±0.01	0.85±0.04	0.89±0.05	
	AdamW [23]	1	0.95±0.03	0.86±0.21	0.70±0.27	0.74±0.19	0.90±0.11	0.82±0.15
		2	0.96±0.03	0.80±0.44	0.60±0.37	0.67±0.39	0.82±0.19	
		3	0.94±0.02	0.83±0.23	0.50±0.17	0.59±0.17	0.76±0.16	
		4	0.94±0.02	0.67±0.41	0.55±0.32	0.58±0.33	0.78±0.21	
		5	0.95±0.03	0.75±0.43	0.60±0.34	0.66±0.38	0.85±0.06	
ResNet [18]	Adam [22]	1	0.97±0.02	1.00±0.00	0.75±0.12	0.85±0.05	0.88±0.02	0.92±0.08
		2	0.98±0.01	0.84±0.08	1.00±0.00	0.91±0.04	0.97±0.05	
		3	0.94±0.01	0.81±0.17	0.55±0.11	0.64±0.07	0.84±0.09	
		4	0.97±0.01	0.95±0.11	0.80±0.11	0.86±0.08	0.97±0.02	
		5	0.97±0.01	1.00±0.00	0.76±0.08	0.86±0.06	0.91±0.08	
	AdamW [23]	1	0.97±0.01	1.00±0.00	0.75±0.12	0.85±0.05	0.83±0.04	0.92±0.07
		2	0.96±0.01	0.74±0.07	1.00±0.00	0.85±0.04	0.96±0.01	
		3	0.95±0.03	0.88±0.24	0.70±0.20	0.73±0.11	0.88±0.09	
		4	0.98±0.01	0.92±0.10	0.90±0.13	0.89±0.05	0.98±0.01	
		5	0.95±0.00	0.87±0.14	0.75±0.19	0.78±0.04	0.91±0.05	
DenseNet [19]	Adam [22]	1	0.93±0.01	0.74±0.22	0.66±0.28	0.65±0.07	0.89±0.04	0.86±0.09
		2	0.96±0.01	0.91±0.13	0.66±0.13	0.75±0.09	0.87±0.06	
		3	0.91±0.02	0.80±0.34	0.33±0.12	0.41±0.02	0.71±0.04	
		4	0.94±0.01	0.69±0.10	0.83±0.14	0.74±0.13	0.94±0.03	
		5	0.94±0.01	0.91±0.14	0.60±0.00	0.72±0.48	0.88±0.09	
	AdamW [23]	1	0.89±0.01	0.47±0.04	0.66±0.14	0.54±0.05	0.85±0.06	0.88±0.06
		2	0.94±0.01	0.86±0.23	0.58±0.14	0.66±0.00	0.88±0.03	
		3	0.91±0.02	0.58±0.13	0.58±0.14	0.56±0.01	0.82±0.08	
		4	0.95±0.01	0.75±0.03	0.75±0.05	0.75±0.04	0.95±0.01	
		5	0.94±0.01	1.00±0.00	0.53±0.11	0.69±0.10	0.88±0.05	
ViT [20]	Adam [22]	1	0.89±0.02	0.48±0.15	0.50±0.00	0.48±0.07	0.74±0.15	0.74±0.15
		2	0.95±0.01	1.00±0.00	0.53±0.18	0.66±0.09	0.75±0.14	
		3	0.96±0.01	1.00±0.00	0.66±0.14	0.79±0.01	0.89±0.11	
		4	0.94±0.02	1.00±0.00	0.41±0.28	0.55±0.26	0.57±0.17	
		5	0.92±0.01	0.07±0.08	0.60±0.00	0.64±0.03	0.75±0.04	
	AdamW [23]	1	0.93±0.01	0.88±0.19	0.41±0.14	0.54±0.13	0.69±0.08	0.77±0.10
		2	0.96±0.01	1.00±0.00	0.58±0.14	0.73±0.10	0.81±0.15	
		3	0.95±0.01	0.83±0.14	0.66±0.14	0.72±0.04	0.89±0.06	
		4	0.95±0.01	1.00±0.00	0.53±0.18	0.66±0.02	0.75±0.04	
		5	0.91±0.02	0.72±0.25	0.40±0.00	0.50±0.06	0.72±0.02	

**Fig 3:**
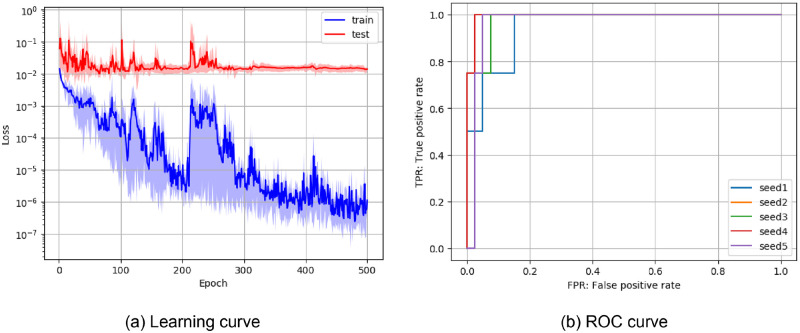
Average learning and ROC curves for subluxation classification. Both results were obtained from the training of ResNet with Adam optimizer and Dataset 2.

Fig 3 shows the training results of ResNet trained with Dataset 2. Fig 3(a) shows the learning curve. The horizontal axis represents the learning progress, and the vertical axis represents the loss value. The learning curve represents the mean and standard deviation across five trials. It can be confirmed that the learning of all four models converged. Fig 3(b) shows ROC curve. The horizontal axis represents the true positive rate, and the vertical axis represents the false positive rate. Although some variation in the performance of the trained model was confirmed, generally good results were obtained.

It can be said that this result was good because mTSS depends on the skill of the doctor. Although images with subluxation findings accounted for approximately only 10% of the entire training dataset, the model could predict subluxation with greater accuracy than ankylosis described in the next section. This may be because the shape of the hands or finger posture of patients with subluxation changes significantly compared to that of patients without subluxation.

**Fig 4:**
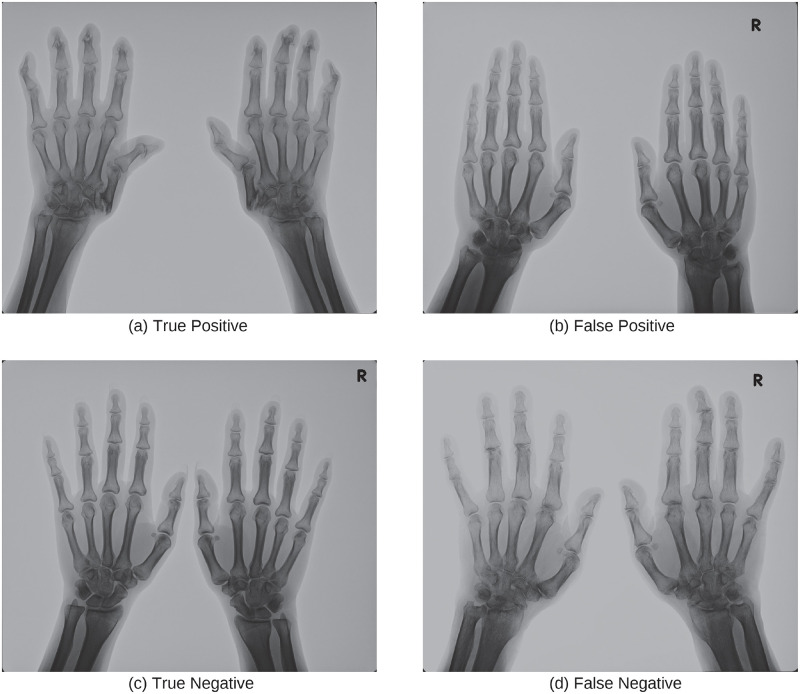
Examples of X-ray images in subluxation dataset. The caption of each image indicates the prediction result.

Fig 4 shows typical examples of true positive, false positive, true negative, and false negative in the classification of subluxation with the trained ResNet. Looking at the examples of DNN models making incorrect decisions (Fig 4d), the model seems to have responded to distal interphalangeal (DIP) joints that are not subject to mTSS. Another false example (Fig 4b) suggests that even a well-trained rheumatologist might find it difficult to make the right decision. Since human judgments vary from person to person, the judgments of mTSS were decided based on an agreement between doctors. In the case of machine learning, combining the output of multiple models usually improves the accuracy (ensemble learning). Therefore, a system that combines the results of machine learning models and the judgment of doctors is suggested [9].

### Ankylosis classification

**Fig 5:**
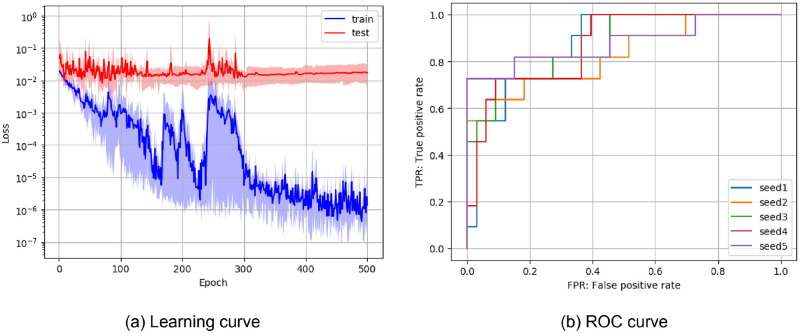
Average learning and ROC curves for ankylosis classification. Both results were obtained from training ResNet with Adam optimizer and Dataset 2.

Table 2 shows the classification results of wrist ankylosis. Similar to the results of subluxation classification, ResNet using Adam optimizer showed the best performance. For averaged results in the overall dataset, accuracy was 0.89±0.04, precision was 0.77±0.15, recall was 0.71±0.13, F1 Value was 0.72±0.09, and AUC was 0.85±0.07. Although the ankylosis classification was less accurate than the subluxation classification, the AUC was 0.85 or higher in both classification tasks. In addition, the learning and ROC curves of ResNet trained with Dataset 2 (Fig 5) also showed the same trend as the subluxation classification. Together with the results of the previous subsection, these results demonstrate the effectiveness of the DNN-based classification method.

**Table 2:** Results of ankylosis classification.

Model	Optimizer	Dataset	Accuracy	Precision	Recall	F1 Value	AUC	Average AUC
AlexNet [2]	Adam [22]	1	0.93±0.07	0.70±0.26	0.84±0.21	0.76±0.24	0.91±0.10	0.80±0.10
		2	0.86±0.04	0.77±0.17	0.70±0.07	0.72±0.04	0.80±0.02	
		3	0.81±0.09	0.51±0.37	0.55±0.32	0.50±0.29	0.82±0.05	
		4	0.87±0.07	0.79±0.25	0.55±0.11	0.62±0.13	0.76±0.08	
		5	0.82±0.04	0.54±0.32	0.44±0.25	0.48±0.27	0.69±0.11	
	AdamW [23]	1	0.96±0.02	0.90±0.09	0.84±0.26	0.83±0.14	0.91±0.14	0.80±0.12
		2	0.88±0.05	0.81±0.13	0.70±0.13	0.75±0.12	0.81±0.06	
		3	0.87±0.05	0.66±0.40	0.55±0.32	0.59±0.33	0.81±0.06	
		4	0.86±0.05	0.65±0.15	0.65±0.05	0.64±0.08	0.81±0.11	
		5	0.80±0.02	0.52±0.31	0.38±0.24	0.42±0.24	0.65±0.08	
ResNet [18]	Adam [22]	1	0.97±0.01	0.96±0.07	0.80±0.14	0.86±0.06	0.94±0.04	0.85±0.07
		2	0.89±0.03	0.79±0.13	0.80±0.07	0.78±0.04	0.88±0.01	
		3	0.86±0.03	0.69±0.18	0.73±0.12	0.68±0.02	0.83±0.04	
		4	0.87±0.02	0.70±0.09	0.60±0.13	0.63±0.07	0.79±0.06	
		5	0.85±0.03	0.71±0.11	0.64±0.05	0.66±0.05	0.80±0.05	
	AdamW [23]	1	0.96±0.01	0.94±0.12	0.80±0.14	0.85±0.06	0.89±0.09	0.84±0.07
		2	0.89±0.02	0.84±0.12	0.74±0.07	0.78±0.02	0.88±0.01	
		3	0.85±0.04	0.65±0.14	0.75±0.14	0.68±0.07	0.80±0.04	
		4	0.89±0.02	0.77±0.14	0.60±0.10	0.66±0.06	0.75±0.06	
		5	0.84±0.03	0.68±0.12	0.68±0.10	0.66±0.03	0.85±0.03	
DenseNet [19]	Adam [22]	1	0.96±0.01	0.79±0.06	0.99±0.01	0.88±0.04	0.97±0.01	0.81±0.11
		2	0.76±0.07	0.57±0.13	0.66±0.22	0.58±0.02	0.75±0.02	
		3	0.88±0.02	0.74±0.12	0.74±0.12	0.72±0.02	0.89±0.02	
		4	0.85±0.01	0.66±0.08	0.45±0.07	0.53±0.03	0.70±0.04	
		5	0.82±0.03	0.65±0.15	0.60±0.10	0.61±0.01	0.71±0.04	
	AdamW [23]	1	0.96±0.01	0.86±0.11	0.80±0.01	0.82±0.05	0.91±0.04	0.79±0.10
		2	0.70±0.09	0.48±0.13	0.81±0.15	0.58±0.04	0.75±0.02	
		3	0.89±0.01	0.71±0.05	0.81±0.06	0.75±0.02	0.88±0.04	
		4	0.89±0.01	0.93±0.11	0.45±0.07	0.60±0.06	0.69±0.05	
		5	0.83±0.03	0.69±0.14	0.56±0.11	0.60±0.01	0.71±0.01	
ViT [20]	Adam [22]	1	0.93±0.01	0.88±0.19	0.60±0.20	0.68±0.09	0.79±0.08	0.73±0.07
		2	0.79±0.03	0.61±0.10	0.57±0.10	0.58±0.04	0.75±0.07	
		3	0.84±0.01	0.66±0.01	0.44±0.01	0.53±0.01	0.67±0.02	
		4	0.81±0.01	0.48±0.03	0.41±0.07	0.44±0.02	0.65±0.03	
		5	0.85±0.01	0.78±0.19	0.53±0.15	0.60±0.05	0.80±0.02	
	AdamW [23]	1	0.92±0.01	0.70±0.08	0.60±0.01	0.64±0.03	0.84±0.06	0.72±0.11
		2	0.76±0.05	0.56±0.14	0.48±0.05	0.41±0.03	0.67±0.06	
		3	0.86±0.01	0.80±0.01	0.44±0.01	0.47±0.01	0.70±0.13	
		4	0.82±0.01	0.55±0.09	0.33±0.07	0.40±0.03	0.62±0.10	
		5	0.85±0.01	0.76±0.20	0.56±0.15	0.62±0.04	0.75±0.09	

**Fig 6:**
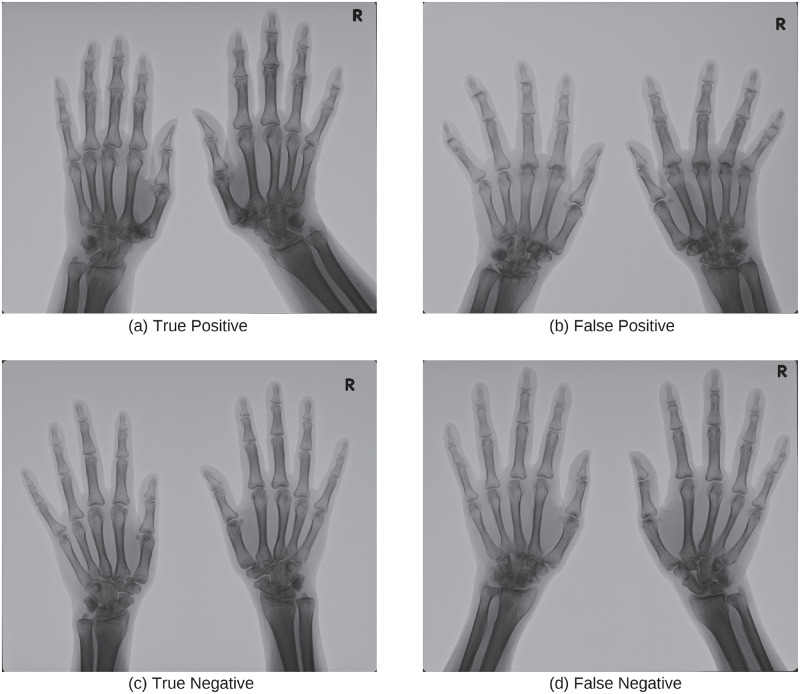
Examples of X-ray images in ankylosis dataset. The caption of each image indicates the prediction result.

Fig 6 shows typical examples of true positive, false positive, true negative, and false negative in classifying X-ray images with ankylosis findings using trained ResNet. The result shows that the classification accuracy (true/false positive) of X-ray images with ankylosis findings was particularly low. This may be because the area of the image which represents ankylosis findings is about tens of pixels square, which is very small compared to the size of the input image. This may be solved by increasing the resolution of the input image or introducing an attention mechanism [21] that emphasizes important parts in the image.

#### Visualizing contributing parts of input image

**Fig 7:**
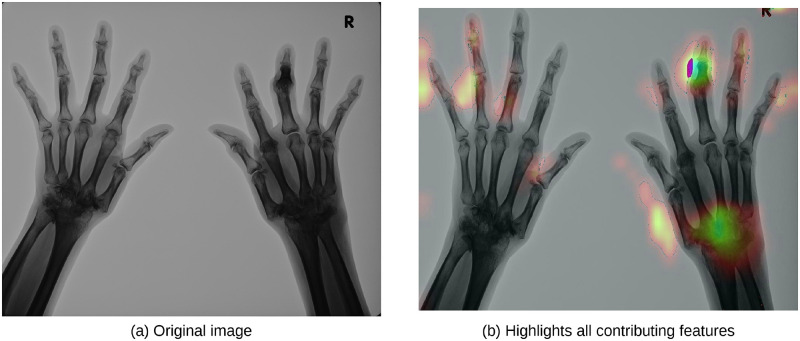
Visualized parts contributing to the prediction result using Grad-CAM. The contribution to the model increases along with the red, yellow, green, and blue scales.

We visualized the parts of the input image that contribute to the output of the model that learned ankylosis classification using Grad-CAM described in the previous section. We visualized ResNet, which showed the best performance in the learning experiments. Fig 7a shows the input image, and Fig 7b shows the image with all contributing features highlighted. The contribution map was obtained from the convolutional layer in Residual Block-3. It was confirmed that the model responded strongly to the position of the PIP joint of the middle finger of the right hand, similar to the subluxation/ankylosis findings by doctors. The joints with suspected findings throughout the dataset (PIP/DIP joint on the left ring finger, list of the right hand) were also captured correctly. This indicates that the model could correctly recognize the image features that determine mTSS.

However, there were some cases where the model focused on areas that had nothing to do with the findings. It was also confirmed that the accuracy of the contribution map decreased from the deep layer to the shallow layer. This may be due to the small amount of training data or variation in the quality of the X-ray images. In future, it will be necessary to increase the size of the image dataset and conduct experiments with high-resolution images.

### Limitations

The proposed method has several issues in building an automatic radiographic scoring system for bone destruction. One is that the proposed model estimated the presence or absence of subluxation/ankylosis from the entire X-ray image of the hand, not each joint. Considering the actual mTSS determination process, it is also necessary to estimate the presence of findings in each joint of the foot as well as the hand. It was difficult to train the model appropriately because the joints of the foot are more complicated than those of the hand, and the number of cases is small. In our experiment, we used the DNN model often used in general image recognition; however, we plan to develop a dedicated model that considers the characteristics of mTSS.

The other limitation is the issue of the quantity of the data samples and the quality of the findings. The size of the dataset used in this study was very small compared to the size of datasets generally used in DNN experiments. We need to collect larger datasets with high-quality annotation. Training is expected to take longer as the scales of the dataset and model increase, but this can be sufficiently improved through the hardware of the computer.

### Related work

In Table 3, we summarized related previous studies where the joint destruction in patients with RA was evaluated using X-ray images by artificial intelligence [25–28]. Miyama et al. [25] developed a classification model for joint space narrowing (JSN) and erosion using VGG-16 in a small number of patients, in which the accuracy of the erosion classification was worse than that of JSN. Ahalya et al. [26] developed a classification model to determine RA from hand X-ray images using GoogLeNet, in which only 10 epochs for pre-trained models and 50 epochs for customized CNN models were used. Wang et al. [27] classified the severity of JSN in the hand using a relatively large amount of images and EfficientNet. Üreten et al. [28] used only hand images to classify RA, OA, and normal images using VGG-16.

**Table 3:** Related previous works and our study.

Study	Year	Dataset	Model	Output	Results	Strength	Weakness
Miyama et al. [25]	2022	226 images of one hand from 40 patients	VGG-16	Presence or absence of erosion or JSN in the PIP-IP, MCP, CMC-M, wrist joints	Erosion: F-value 0.52, PR-AUC 0.54; wrist, F- value 0.84, AUC 0.88, JSN: F- value 0.76, PR-AUC 0.81; wrist, F- value 0.88, AUC 0.93	Classification model for existence of JSN and erosion was developed in a small number of patients.	Small number of patients. No assessment of the degree of JSN and erosion.
Ahalya et al. [26]	2022	100 hand images	GoogLeNet	RA or normal	Classification of RA and normal: Accuracy 0.95, sensitivity 0.95, specificity 0.94	Only 10 epochs for pre-trained models and 50 epochs for customized CNN models were used.	Small number of X-ray images. Wrist joints were not included.
Wang et al. [27]	2022	915 hand images from 400 patients	EfficientNet	Severity of JSN (severe, mild, healthy) in PIP, MCP, and wrist	Classification of JSN: precision 0.88, recall 0.88, F-value 0.88	Classification of the severity of JSN in the hand by a relatively large number of patients.	The accuracy of the classification for each joint is unknown. No evaluation for joint erosion.
Üreten et al. [28]	2022	RA, 368 hand images; OA, 377; normal 333; other 348	VGG-16	RA, OA, or normal	Classification of RA, OA, and normal: accuracy 0.806	Classification of RA, OA, and normal was made solely by hand images.	The extent of bone destruction in RA and OA used in the study is unknown.
Ours	–	216 images of both hands from 216 patients	ResNet	Presence or absence of subluxation or ankylosis in the hands	Subluxation: F-value 0.82, AUC 0.92 Ankylosis: F-value 0.82, AUC 0.92	A model that is capable of detecting subluxation and ankylosis on hand X-ray images was developed.	Relatively small number of patients. No quantification of joint destruction.

AUC, area under the curve; CMC, carpometacarpal joint; CMC-M, carpometacarpal joint of the thumb and multangular; CNN, convolutional neural network; IP, interphalangeal; JSN, joint space narrowing; MCP, metacarpophalangeal; NA, not available; OA, osteoarthritis; PIP, proximal interphalangeal; PR, precision-recall, RA, rheumatoid arthritis.

## Conclusion

In this study, we proposed a DNN model for subluxation/ankylosis classification as the first step for an automatic radiographic scoring system. We collected the X-ray image data by developing a dedicated annotation tool for mTSS. As a result of learning experiments using some DNNs, (AlexNet, ResNet, DenseNet, and ViT) models that are capable of detecting subluxation and ankylosis on hand X-ray images with a relatively small number of samples were constructed. ResNet showed the best performance in both subluxation/ankylosis classification tasks. In addition, we visualized the contributing parts of the input images to the output of the model that learned ankylosis classification using Grad-CAM. The results indicated that the model could correctly recognize the image features that determine mTSS. In conclusion, we provided the data collection and model training schemes for mTSS prediction and showed an important contribution to building an automated estimating system. In future, we plan to extend this study, and our other study [29, 30] to automatically estimate joint destruction more accurately.
